# Stiffness Regulates the Morphology, Adhesion, Proliferation, and Osteogenic Differentiation of Maxillary Schneiderian Sinus Membrane-Derived Stem Cells

**DOI:** 10.1155/2021/8868004

**Published:** 2021-07-08

**Authors:** Yiping Liu, Jia Wang, Peisong Zhai, Sicong Ren, Zhanqi Wang, Peixuan Peng, Liuyi Du, Lisha Li, Yidi Zhang, Yanmin Zhou

**Affiliations:** ^1^Department of Oral Implantology, School and Hospital of Stomatology, Jilin University, Changchun 130021, China; ^2^The Key Laboratory of Pathobiology, Ministry of Education, Norman Bethune Medical College, Jilin University, Changchun 130021, China

## Abstract

Recent studies, which aim to optimize maxillary sinus augmentation, have paid significant attention exploring osteogenic potential of maxillary Schneiderian sinus membrane-derived cells (MSSM-derived cells). However, it remains unclear that how MSSM-derived cells could respond to niche's biomechanical properties. Herein, this study investigated the possible effects of substrate stiffness on rMSSM-derived stem cell fate. Initially, rMSSM-derived stem cells with multiple differentiation potential were successfully obtained. We then fabricated polyacrylamide substrates with varied stiffness ranging from 13 to 68 kPa to modulate the mechanical environment of rMSSM-derived stem cells. A larger cell spreading area and increased proliferation of rMSSM-derived stem cells were found on the stiffer substrates. Similarly, cells became more adhesive as their stiffness increased. Furthermore, the higher stiffness facilitated osteogenic differentiation of rMSSM-derived stem cells. Overall, our results indicated that increase in stiffness could mediate behaviors of rMSSM-derived stem cells, which may serve as a guide in future research to design novel biomaterials for maxillary sinus augmentation.

## 1. Introduction

Millions of lost teeth need dental implant treatments each year [1]. However, the insufficient residual bone volume in posterior maxilla secondary to maxillary sinus pneumatization could significantly hamper implant prosthesis placement in this area [2, 3]. To date, sinus augmentation has been proved effective for augmenting alveolar bone before implant placement in these regions [4]. However, the mechanisms of osteogenesis after sinus augmentation are still inadequately analyzed. Furthermore, there have been many reports describing the applications of various graft materials with different constructures as well as composition and compared their capacities to enhance osteogenesis during sinus augmentation [5, 6]; yet, few of them have investigated from the perspective of signals delivered by extracellular matrices (ECM). Thus, analyzing the potential relationship between cells and signals of ECM might aid in development of novel clinical therapy for sinus augmentation.

Recently, findings have demonstrated that cells derived from the maxillary Schneiderian sinus membrane (MSSM) possess osteogenic potential [7–10]. A previous study [7] had reported that MSSM contains mesenchymal osteoprogenitor cells capable of expressing osteogenic markers as well as producing osteopontin (OPN). According to Srouji et al. [8], MSSM-derived cells could express diverse markers of osteoprogenitor cells, undergo osteogenic differentiation in vitro and form new bone after transplantation in vivo. Additionally, to promote both the quality and quantity of new bone of cultured MSSM-derived cells, various materials such as gelatin scaffolds and simvastatin were utilized [9, 10]. However, the influence of ECM signals on MSSM-derived cells remained poorly understood.

Stiffness, among many biophysical signals, could potentially alter cell spreading, proliferation, and stem cell fate [11–16], via a mechanical interaction [13]. For example, cornea epithelial stem cells on lower stiffness showed higher proliferation, higher stratification, and reduced migration capabilities [17]. Besides, another study on bone marrow-derived stem cells demonstrated that low-stiffness gels promoted cell proliferation, whereas high-stiffness gels facilitated cell osteogenic differentiation [11]. The stiffness induced behavioral differences among the different kinds of reproducible cells, including dental pulp stem cells (DPSCs) and periodontal ligament stem cells (PDLSCs) within the dental area [18, 19], and others such as valve interstitial cells (VICs) [20] and oral squamous cell carcinoma cells [21] have been investigated, thus bringing more significance to this parameter in tissue engineering and regenerative therapies. It has been established based on the various previous studies on mesenchymal stem cells (MSCs) that the softer substrate matrix is suitable to induce neurogenesis and adipogenesis, while stiffer matrix tended to induce myogenesis chondrogenesis and osteogenesis [14, 22–24]. Moreover, as for other cells, upregulated osteogenic markers in DPSCs, PDLSCs, and VICs on stiffer substrates were also reported [18–20]. These findings clearly indicated a stiffness-associated osteogenic differentiation incentive among the abovementioned cells.

With regard to maxillary sinus elevation results, the bone formation quality plays a decisive role. Given the lack of the study on stiffness-mediated rMSSM-derived stem cells behavioral differences, we conducted this research to investigate the potential behavioral response of rMSSM-derived stem cells to substrate stiffness. It has been found that partly due to size and ease of handling [25], rabbit was one of the most commonly used animals for the maxillary sinus elevation model of medical research studies [5, 26, 27]. Therefore, the present study used rMSSM-derived stem cells of rabbits for the various in vitro experiments. Herein, we have observed that the cellular behaviors of rMSSM-derived stem cells including morphology, adhesion, proliferation, and osteoblastic differentiation were significantly altered by substrate stiffness. Meanwhile, the current study may provide a new method to promote osteogenic ability of rMSSM-derived stem cells, thus guiding the material design in maxillary sinus augmentation.

## 2. Materials and Methods

### 2.1. Isolation and Culture of rMSSM-Derived Stem Cells

rMSSM-derived stem cells were isolated from 6-month-old Japanese white rabbits (*n* = 6) as previously described with slight modifications [7, 8]. Two pieces of tissues were isolated from each donor for the experiments. All experiment procedures were approved by the Institutional Animal Care and Use Committee of Jilin University. Briefly, MSSM tissues derived from maxillary sinus of rabbits were washed with phosphate-buffered saline (PBS, Hyclone, Logan, UT) containing 10% penicillin/streptomycin (P/S, Hyclone, Logan, UT) very quickly and then were thoroughly washed with PBS containing 1% P/S. To separate the epithelial lining, tissues were digested with 1 U/mL dispase I solution (Sigma-Aldrich, St Louis, MO) in PBS for 1 h at 37°C. The epithelial cells were discarded, and the remaining tissues were cut into small pieces by blade and incubated with 200 U/mL collagenase solution (Sigma, Lakewood, NJ) in PBS for 12 h at 4°C. The cells were obtained by filtration using a 70 *μ*m cell strainer (BD biosciences, San Jose, CA). The isolated cells were seeded into 25 cm^2^ tissue culture flasks (WHB, shanghai, China) with alpha minimum essential medium (*α*-MEM, Hyclone, Logan, UT) containing 10% fetal bovine serum (FBS, Hyclone, Logan, UT) and 1% P/S. The cultures were maintained at 37°C in a humidified atmosphere with 5% CO_2_, and the medium was changed twice a week in order to remove the nonadherent cells. When the cell population reached 80-90%, the primary cells were passaged using 0.25% trypsin enzyme (Hyclone, Logan, UT), followed by centrifugation, resuspension, and reseeding up to 2-4 passages. For sphere formation, the cells were plated at clonal density (<1,000 cells cm^−2^) in ultra-low attachment surface plates (Corning, Corning, NY). The cells were observed under inverted phase contrast microscope (Olympus, Tokyo, Japan).

### 2.2. Flow Cytometric Analysis

The detection of various surface markers was performed by flow cytometric analysis. The cells were digested with 0.25% trypsin, centrifuged at 600 × g, 4°C for 5 min, and washed with PBS for three times. The cell suspension, at a density of 1 × 10^6^ cells/mL, was incubated with primary antibodies for 1 h, on ice and in dark (Anti-rat CD29-FITC, Biolegend, San Diego, CA; anti-human CD34-FITC, Thermo Fisher scientific, Fremont, CA; anti-rat CD90- PE-cy7, BD biosciences, San Jose, CA; anti-rabbit CD45, Bio-Rad, Kidlington, UK; anti-rabbit CD44, Bio-Rad, Kidlington, UK) and then washed with PBS for three times. The incubation with secondary antibodies of CD44 and CD45 antibodies (anti-mouse IgG-PE, Miltenyi Biotec, Bergisch Gladbach, Germany) was carried out according to the same procedure as used for the primary antibodies. The cell suspension incubated with PBS was used as a negative control. After incubation and washing with PBS, approximately 1 × 10^6^ of cells was resuspended in 300 *μ*L PBS and examined by FACSCalibur flow cytometer (BD Biosciences, San Jose, CA). The cells were gated according to their forward (FSC) and side scattered (SSC) properties during the analysis.

### 2.3. Osteogenic, Adipogenic, and Chondrogenic Differentiations of rMSSM-Derived Stem Cells

rMSSM-derived stem cells were cultured in osteogenic medium (*α*-MEM medium containing 10% FBS, 10 mM *β*-glycerophosphate, 50 *μ*g/mL ascorbic acid, 1% P/S, and 10 nM dexamethasone) and adipogenic medium (*α*-MEM medium containing 10% FBS, 0.5 mM 3-isobutylene-1-methylxanthine, 100 nM dexamethasone, 10 *μ*g/mL insulin, 1% P/S, and 50 mM indomethacin) to induce osteogenic and adipogenic differentiations. For chondrogenic differentiations, cells (5 × 10^5^) were resuspended and precipitated in 0.5 mL of commercially differentiation media kits (Cyagen, Suzhou, China) to form high-density cartilage pellets. The pellets were incubated in an incubtator with 5% CO_2_ at 37°C. Medium were replaced at a 3-day cycle. Alizarin Red and Oil Red O staining were carried out after 4 weeks and 2 weeks of osteogenic and adipogenic culture, respectively, to verify the stemness of these cells. Cells for staining were seeded in 6-well plates at a density of 1 × 10^5^ cells/well and were fixed with 4% paraformaldehyde for 10-15 min. 1% Alizarin Red S solution (Cyagen, Suzhou, China) and Oil Red O (Cyagen, Suzhou, China) were applied for 15 min at room temperature. For chondrocyte differentiation, the pellets were fixed with 4% paraformaldehyde for 3 days after 3 weeks of culture, embedded in paraffin after being dehydrated, cut into 5 *μ* m-thick sections, and stained with Alcian blue after being dewaxed with xylene and dehydrated with alcohol (Cyagen, Suzhou, China). Cells were observed under inverted phase contrast microscope (Olympus, Tokyo, Japan).

### 2.4. Fabrication of Polyacrylamide Substrates

Polyacrylamide substrates with varying stiffness were fabricated as described previously [28]. Briefly, 8% acrylamide and 0.1%, 0.5%, and 0.7% *bis*-acrylamide were mixed and then polymerized with 10% ammonium persulfate (AP; Sigma-Aldrich, St Louis, USA) and tetramethylethylenediamine (TEMED; DingGuo, beijing, China). The mixture was thereafter transferred to 24-well and 6-well plates by using coverslips previously treated with 3-aminopropyltrimethoxysilane (Sigma-Aldrich, St Louis, MO) and 0.5% glutaraldehyde (Sigma-Aldrich, St Louis, MO). After that, the mixture was coated with 0.2 mg/mL N-sulfosuccinyimidyl-6-(4′-acidosis-2′nitrophenylamino) hexanoate (sulfa-SANPAH; ThermoScientific, Waltham, MA) dissolved in 10 mM HEPES (pH 8.5) and was exposed in 365 nm ultraviolet light for 70 min to facilitate photoactivation. The polyacrylamide was washed with PBS to remove excess reagent and then incubated in 2 *μ*g/cm^2^ fibronectin solution (Sigma-Aldrich, St Louis, MO) overnight at 4°C. Young's modulus of each sample was thereafter measured with a biomechanical testing machine under contact load at a strain rate of 0.5 mm/s [28].

### 2.5. Cell Proliferation Analysis

The cell proliferation on different stiffness of polyacrylamide substrates was analyzed via Cell Counting Kit-8 assay (CCK-8; NCM Biotech, Suzhou, China). Briefly, the cells were resuspended, counted, and seeded at a density of 8000 per well onto 24-well plates, in which gel slide with different stiffness had been placed. After culturing the cells for 1, 3, 5, and 7 days, the CCK-8 assay was carried out following the manufacturer's instructions. CCK-8 was added, and the samples were kept for 2 h in the incubator. The degree of absorbance of CCK-8 solution at 450 nm was analyzed by a Synergy HT spectrophotometer (Bio Tek Instruments, Winooski, VT).

### 2.6. Immunofluorescence

After a 12-hour culture in different stiffness, the culture medium was removed, and the cells were washed with PBS for three times. The cells were then fixed by 4% paraformaldehyde for 30 min at 4°C and washed, ready for Fluorescein isothiocyanate- (FITC, Sigma-Aldrich, St Louis, MO-) conjugated phalloidin and 4′,6-diamidino-2-phenylindole (DAPI, Sigma-Aldrich, St Louis, MO) staining. 10 *μ*g/mL FITC-phalloidin and 1 *μ*g/mL DAPI were applied in sequence, each for 10 min at room temperature and in darkness. The staining agents were removed altogether, and the samples were washed before observed under confocal microscopy (Olympus FV3000, Tokyo, Japan; lamp: U-HGLGPS; microscope: IX83; controller components: I3-TPC, U-MCZ, CBH Control Box; power supply unit: FV31-SU-P). To measure the cell area, ImageJ software was used to analyze FITC staining. The aspect ratio of cell is the ratio of major to minor axis which was also computed from the threshold binary image of the cell using ImageJ. The cells were subjected to immunofluorescence using the primary antibodies of vinculin and osteopontin after 3 days of incubation. The samples on the different substrates were rinsed and then fixed with 4% paraformaldehyde. To permeabilize the cells, 0.1% Triton X-100 was applied for 15 min. Thereafter, the cells were blocked with 1% bovine serum albumin (BSA) for 1 h at room temperature and then incubated with the primary antibody (Santa Cruz Biotechnology Inc., Dallas, TX) overnight at 4°C. The cells were then incubated with the secondary antibody (Beyotime, shanghai, China) for 1 h. Thereafter, the counterstaining with DAPI (Sigma-Aldrich, St Louis, MO) for 10 min was performed. The samples were observed under fluorescent microscope (Olympus, Tokyo, Japan; mercury lamp: U-RFL-T; microscope: IX73). ImageJ was used for quantification of relative fluorescence intensity of vinculin and OPN in the different groups. The quantification was performed by measuring three images in the different wells and not just one image/well.

### 2.7. Real-Time Quantitative PCR

rMSSM-derived stem cells were resuspended, counted, and seeded onto the FN-coated substrates at a density of 8 × 10^3^ cell/cm^2^ and cultured in the normal *α*-MEM medium. The cells were induced to differentiate into osteoblasts-like cells by converting to osteogenic medium. The medium was changed every 3 days. Total RNA of rMSSM-derived stem cells was extracted with TRIZOL (TAKARA, kusatsu, Japan), and reverse transcription was performed using TAKARA Reverse Transcriptase kit (TAKARA, Osaka, Japan) following the manufacturer's instructions. The concentration and quality of RNA were determined by Thermo NANODROP 2000c (Thermo Fisher scientific, Fremont, CA). Thereafter, the polymerase chain reaction (PCR) was performed using PrimeScript™ RT-PCR kit (TaKaRa, Tokyo, Japan) and Applied Biosystems 7300 (ThermoScientific, Waltham, MA) according to the instructions. The cycling conditions used for PCR reaction consisted of 95°C for 30 seconds, followed by 40 cycles of 95°C for 5 seconds, and 60°C for 31 seconds. All the primer sequences of the various markers analyzed have been listed in Table 1. The data was analyzed by the 2^-∆∆Ct^ method and normalized to that of the glyceraldehyde-3-phosphate dehydrogenase (GAPDH) expression. Each experiment was carried out in triplicate.

### 2.8. Statistical Analysis

All the values have been presented as mean ± standard deviation (SD) from at least three independent experiments. The differences were analyzed with SPSS 26.0 (IBM) using one-way ANOVA and posthoc Tukey's test. The differences were considered as statistically significant when *P* < 0.05.

## 3. Results

### 3.1. The Characterization of rMSSM-Derived Stem Cells

**Figure 1:**
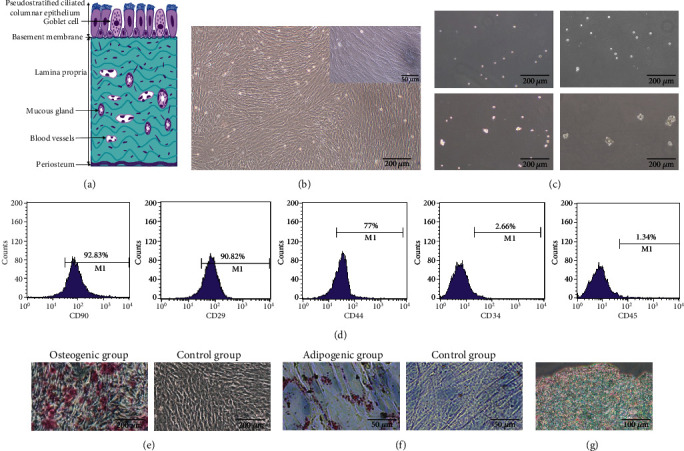
Schematic draft of the maxillary Schneiderian sinus membrane (MSSM) and identification of the MSSM-derived stem cells. (a) Schematic representation showing the pseudostratified epithelium, lamina propria, and periosteum-like components of the MSSM. (b) Morphological appearance of second-passage rMSSM-derived stem cells. (c) Ability to form spheres of rMSSM-derived stem cells cultured in nonadherent conditions on days 1, 2, 4, and 5. Scale bars are 200 *μ*m. (d) Cell surface markers of rMSSM-derived stem cells evaluated through cytometric flow analysis. (e) Differentiation of rMSSM-derived stem cells into osteoblasts stained by alizarin red S. Scale bars are 200 *μ*m. (f) Differentiation of rMSSM-derived stem cells into adipocytes stained by Oil Red O. Scale bars are 50 *μ*m. (g) Differentiation of rMSSM-derived stem cells into chondrogenic pellets stained by Alcian Blue. Scale bars are 100 *μ*m.

MSSM, a bilaminar membrane including ciliated columnar epithelial and periosteum [8], was successfully separated from maxillary sinus of rabbits (Figure 1(a)). The cells could successfully grow out of the MSSM tissues after 3-6 days of primary culture. Moreover, the cells principally formed bipolar spindle-like and fibroblast-like cells after they have grown to passage 2 (Figure 1(b)). The mesenchymal stem cell properties of rMSSM-derived stem cells were confirmed by carrying out the colony formation assay, determination of various mesenchymal-associated surface markers, and the multidirectional differentiation capability. Sphere-forming assays have been widely used to evaluate the capacity of self-renewal in vitro [29, 30]. The cell clonal spheres became larger from day1 to day 5, which clearly reflected their self-renewal capacity (Figure 1(c)). The expression of different surface markers of rMSSM-derived stem cells (P3) was analyzed by flow cytometric analysis to identify rMSSM-derived stem cells. Mesenchymal-associated antigens such as CD29 (92.79 ± 4.3%, *n* = 3), CD90 (93.33 ± 3.1%), and CD44 (77.50 ± 2.6%) expressions were observed to be positive, while hematopoietic markers such as CD34 (3.31 ± 2.0%) and CD45 (3.40 ± 3.1%) were negative (Figure 1(d)). Osteogenic, adipogenic, and chondrogenic differentiation potential was demonstrated by using Alizarin Red S, Oil Red O, and Alcian blue staining after relative inductive culture. It was noted that dense mineral deposits presented in cells in Alizarin Red S staining after a 3-week osteogenic culture as compared with the control group (Figure 1(e)). In Oil Red O staining, meanwhile, cells exhibited numerous Oil Red O-positive lipid globules after 2 weeks of adipogenic induction compared with the control group (Figure 1(f)). Moreover, rMSSM-derived stem cells were able to effectively differentiate into chondrocytes (Figure 1(g)), but the control groups without chondrogenic induction were not able to form the cell pellets. These results clearly demonstrate that strategies used to obtain rMSSM-derived stem cells were successful.

### 3.2. Effect of Substrate Stiffness on Cell Morphological Changes

**Figure 2:**
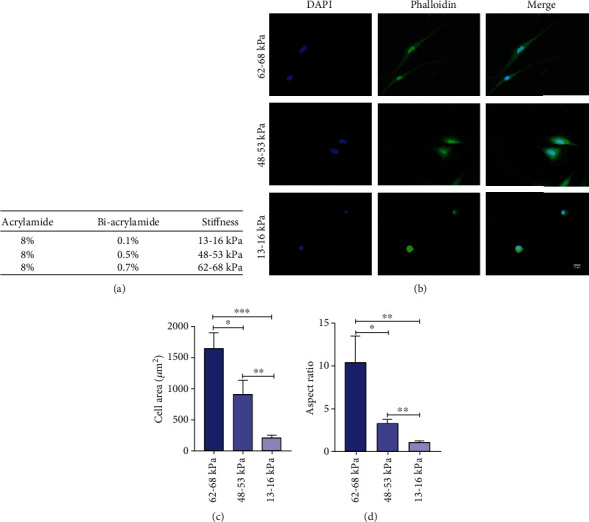
Stiffness altered the organization of cytoskeletal filaments. (a) Substrates were fabricated by 8% acrylamide with 0.1%, 0.5%, and 0.7% bis-acrylamide. (b) Immunofluorescence staining of cytoskeleton by DAPI (blue) and FITC-phalloidin (green) for rMSSM-derived stem cells. Scale bars are 15 *μ*m. (c, d) Quantification of morphological changes of rMSSM-derived stem cells cultured on various stiffness substrates. The statistics were representative of three independent samples (*n* = 3). ^∗^*P* < .05, ^∗∗^*P* < .01, ^∗∗∗^*P* < .001.

The stiffness of polyacrylamide was modified by adjusting the concentrations of bisacrylamide. The stiffness of three kinds of gels was about 13-16, 48-53, and 62-68 kPa (Figure 2(a)). After 12 h of culture, FITC-phalloidin staining was performed to evaluate the potential cytoskeleton differences of rMSSM-derived stem cells on substrates with different stiffness (Figures 2(b), 2(c), and 2(d)). According to fluorescence images of actin filaments, the cells in 62-68 kPa ECM appeared more stretched out, spindle-shaped, and contained linear actin filaments. As the substrate stiffness decreased, the cells on 48-53 kPa ECM adapted a polygonal shape. Moreover, cells on ECM with the lowest stiffness presented a round-shaped and compact morphology with the actin fibers surrounding the nucleus (Figure 2(b)). Moreover, the cell spreading area and aspect ratio were observed to increase significantly on the stiffer substrates (Figures 2(c) and 2(d)).

### 3.3. Regulation of Substrate Stiffness on Vinculin Distribution

**Figure 3:**
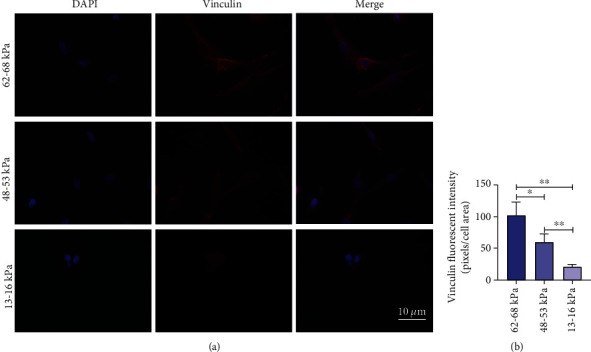
Adhesion protein expression of rMSSM-derived stem cells on different substrate stiffness. (a) Immunofluorescence images showing the changes of vinculin in rMSSM-derived stem cells regulated by substrate stiffness. DAPI (blue) and vinculin (red). (b) Quantification of relative fluorescence intensity of vinculin in different groups. The statistics were representative of three independent samples (*n* = 3). Scale bars are 10 *μ*m. ^∗^*P* < .05, ^∗∗^*P* < .01, ^∗∗∗^*P* < .001.

We also explored vinculin, a key adhesion protein, which mediates cellular functions like adhesion, migration, and mechanosensing by binding focal adhesions (FAs) [31]. The immunofluorescence images showed that the expression of vinculin was stiffness-dependent (Figures 3(a) and 3(b)). In the 62-68 kPa substrates, vinculin distributed more widely, and its expression was brighter than in the softer substrates. However, as the substrates became softer, vinculin showed dense distribution and decreased expression.

### 3.4. Effect of Substrate Stiffness on Proliferation of rMSSM-Derived Stem Cells

**Figure 4:**
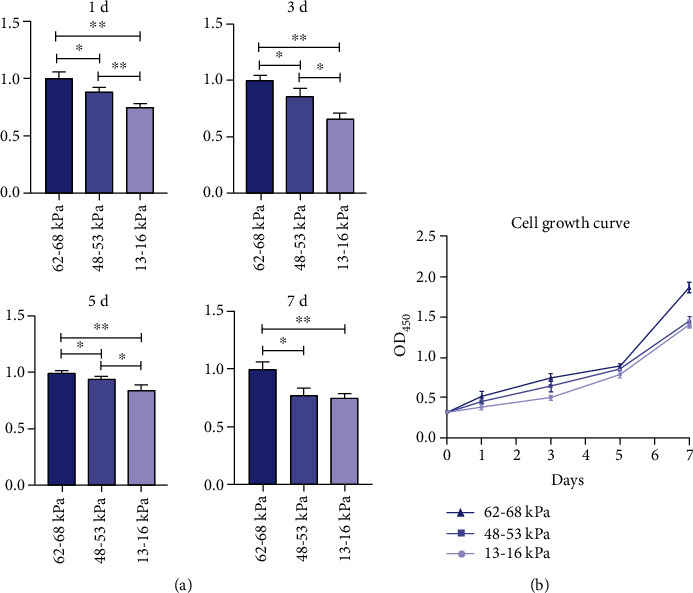
The CCK-8 assay was carried out after 1, 3, 5, and 7 days to assess the proliferation of rMSSM-derived stem cells regulated by variable matrix stiffness. (a) The results of the CCK-8 assay were represented by histogram. Data are normalized to that of 62-68 ECM. (b) Cell growth curve of rMSSM-derived stem cells. The statistics were representative of three independent samples (*n* = 3). ^∗^*P* < .05, ^∗∗^*P* < .01.

The cell proliferation on substrates with increased stiffness was investigated via the CCK-8 assay on the first, third, fifth, and seventh days of culture. All data were normalized to that of 62-68 kPa ECM to better compare the possible effect of stiffness. The cell proliferation was significantly enhanced on the substrate with the highest stiffness of 62-68 kPa (Figure 4). It also showed on days 1, 3, and 5 that the proliferation was promoted as the substrate stiffness increased. Therefore, a trend that higher substrate stiffness enhances cell proliferation was clearly noticed according to these results.

### 3.5. Regulation of Substrate Stiffness on Osteogenic Differentiation

**Figure 5:**
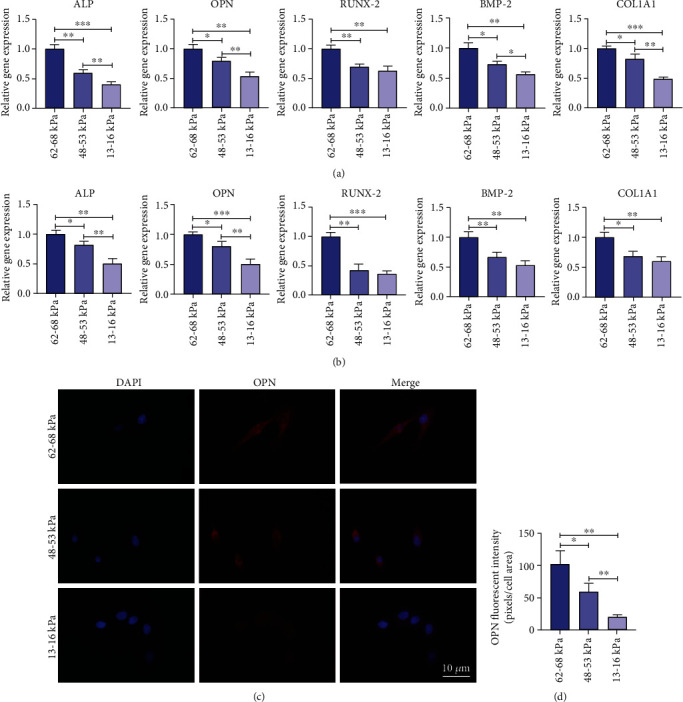
Osteogenic differentiation of rMSSM-derived stem cells on different substrate stiffness. (a) The expression levels of ALP, OPN, RUNX-2, BMP-2, and COL1A1 were detected by qRT-PCR after 3 days. (b) The expression levels of ALP, OPN, RUNX-2, BMP-2, and COL1A1 were detected by qRT-PCR after 7 days. (c) Immunofluorescence images of the OPN expressed in rMSSM-derived stem cells cultured on different stiffness substrates. OPN (red) and DAPI (blue). (d) Quantification of relative fluorescence intensity of OPN in different groups. The statistics were representative of three independent samples (*n* = 3). Scale bars are 10 *μ*m. qRT-PCR results were presented as 2^-∆∆Ct^. ^∗^*P* < .05, ^∗∗^*P* < .01, ^∗∗∗^*P* < .001.

To investigate the role of substrate stiffness in the osteogenic differentiation of rMSSM-derived stem cells, the cells were cultured on the substrates with varying stiffness in the presence of osteogenic medium. We then carried out quantitative real-time PCR (qRT-PCR) assays and immunofluorescence analysis. qRT-PCR results indicated a marked increase in the mRNA expression level of ALP (alkaline phosphatase), OPN (osteopontin), RUNX-2 (runt-related transcription factor-2), BMP-2 (bone morphogenetic protein-2),and COL1A1 (collagen, type I, alpha 1) at both day 3 and day 7 on 62-68 kPa ECM (Figures 5(a) and 5(b)). Additionally, after rMSSM-derived stem cells were cultured for 3 days, the strongest immunofluorescence of OPN on 62-68 kPa ECM was observed (Figures 5(c) and 5(d)).

## 4. Discussion

Our results illustrated that substrate stiffness could alter several important cellular behaviors of rMSSC-derived stem cells. It was found that the stiffer substrates facilitated proliferation, adhesion, and osteoblastic differentiation of cells in the current study. This mechanical-mediated regulation of behavior of rMSSC-derived stem cells has significant implications in bone biology and bone tissue engineering. Here, we fabricated elastic polyacrylamide hydrogels by mixing 8% acrylamide with 0.1%, 0.5%, and 0.7% bisacrylamide, which mimicked cellular biomechanical signal. To facilitate adhesion of rMSSC-derived stem cells [32], the substrates were then coated with fibronectin layer. Synthetic polyacrylamide (PA) hydrogels are cell-repellent [33], and culturing the cells on PA requires the covalent coupling of cell-adhesive matric protein by protein-substrate linker, which includes sulpho-SANPAH as used in the current study [15]. Fibronectin was chosen because it is an essential component of extracellular matrix, and it can participate in all stage of life activities [34]. The cells could adhere to fibronectin-immobilized materials as the cell adhesion molecules can bind to RGD sequence of fibronectin [35]. Moreover, other materials such as collagens have also been used in studies of stiffness, but the cell adhesion on collagen is mainly indirectly mediated by matrix glycoproteins [36]. In addition, adding fibronectin could help smoothen the surface of hydrogels to get rid of the impact of topography on the cell growth [37]. In this study, the PA demonstrated a number of favorable features [28, 37]. First, it was found to be relatively safe and nontoxic. Second, it was reproducible and systematic to control the stiffness of substrate. Third, its porous nature enabled the media penetration to provide the cells a more of the physiological microenvironment.

Substrate stiffness, acting as a vital biomechanical factor, has been reported to modulate cellular functions in previous studies [18, 19, 38]. It has been demonstrated that MSCs favor neuronal differentiation on relatively soft substrates, whereas they undergo osteogenic differentiation as the substrate stiffness increases [14]. Additionally, the cellular behaviors of numerous cells, such as DPSCs, PDLSCs, and human stem cells of apical papilla (hSCAPs) could be similarly regulated by the substrate stiffness [18, 19, 38].

However, little is known about the potential interaction between MSSC-derived stem cells and physical microenvironment. The MSSM is a bilaminar membrane that can attach to the inner wall of maxillary sinus [8]. By elevating MSSM, sinus floor augmentation can provide optimal vertical dimension for implant placement in the posterior maxilla with poor bone quality [39]. A number of previous studies have primarily concentrated on comparing efficacy of the different materials applied in sinus elevation and exploring how to promote osteogenesis of MSSC-derived cells [40–42]. Hitherto, there was little consensus in choosing the ideal graft materials for maxillary sinus augmentation, even though various materials were applied in the previous studies [43–45]. Thus, understanding the correlation of the properties of substrate with cellular behaviors is conducive to ensure the effectiveness of the devices [46]. In this study, soft substrates (13-16 kPa) were found to mimic the elasticity of muscle, whereas the stiffer substrates (48-53 kPa) and hardest (62-68 kPa) substrates could mimic the characteristics of the premineralized bone [11]. We selected this range of substrate stiffness (13-68 kPa) also based on the previous findings that indicated that various cells are sensitive to the stiffness within this range [47–49]. However, the stiffness of human organs varies widely, which possesses moduli from 1 kPa (like brain) to 15 MPa (like mineralized bone tissue) [50]. Thus, other ranges of stiffness like 0.5-50 kPa [47], 1.4-134 kPa [51], and 0.6-2.7 MPa [46] were also applied in the related studies. The larger range of stiffness would be used in our future research.

In our study, we demonstrated that cytoskeleton of rMSSC-derived stem cells changed significantly in response to the varied stiffness. Fibroblast-like and stretched out cells were detected on the 62-68 kPa stiff group, whereas rMSSC-derived stem cells adopted to adipocyte-like shapes and were found to spread poorly on the softer substrates. A similar phenomenon was observed in a previous work by Zhang et al. who reported that osteoblasts were widely stretched out on the stiff group but shrank into small size on the soft substrates [51]. It can be explained that cells favored assembling in rounder morphologies when traction generated by the cells against ECM exceeds constrain stress given by the substrates [51]. The findings in two dimensional settings have clearly indicated the interplay between cell shape and function [52] and have suggested that the functions of rMSSC-derived stem cells may be closely related to the substrate stiffness.

Cell proliferation and differentiation are two key factors involved in regulating the process of tissue regeneration [53]. In our study, an enhanced rate of proliferation of rMSSC-derived stem cells was observed on the stiffer substrates as compared with intermediate or low modulus substrates, which indicated that the quantity of rMSSC-derived stem cells available for regeneration increased. Many studies have also previously corroborated the stiffness-mediated cell proliferation changes. PDLSCs and DPSCs could undergo maximal proliferation on the stiff substrates of 135 kPa [18, 19], whereas the neural stem cells prefer medium stiffness of 3.5 kPa to proliferate [50]. In accordance with previous studies, our work added a new type of cell to the various studies supporting the findings that the cell proliferation could be significantly affected by stiffness. However, a similar proliferation rate was observed on the low and intermediate modulus substrates on day 7 in our work, which may indicate that the relationship between stiffness and proliferation could be possibly nonlinear.

The medium was converted to osteogenic medium when cells were induced to undergo differentiation into osteoblasts-like cells. The osteogenic differentiation ability was measured by analyzing the expression levels of various osteogenic markers, including ALP, OPN, RUNX-2, and BMP-2 as well as COL1A1, and our results clearly demonstrated that rMSSC-derived stem cells took advantage of the stiff substrates to differentiate. However, there was no significant difference in the expression of RUNX-2, BMP-2, and COL1A1 between intermediate and soft groups as shown in 4 of 10 bar graphs (Figure 5), which indicated that rMSSC-derived stem cells could potentially respond strongly to the stiff and soft substrates than to intermediate matrix in terms of osteogenic differentiation. Similarly, previous work by Datko's group has suggested that DPSCs may only show distinct osteogenic differentiation on very stiff substrates (>75 kPa) [54]. Although most studies concluded that an increased stiffness is conducive to osteogenic differentiation, a study, manufacturing wide range of elastic modulus, reported that softer substrates were more conducive to osteogenic differentiation as compared to the stiffer ones [55]. Therefore, optimal stiffness necessary for inducing rMSSC-derived stem cells to bone lineages requires further exploration.

We then investigated the expression of vinculin, a key adhesion protein that could link ECM and cytoskeleton by reinforcing integrin binding [56]. Our results showed that the vinculin expression in the cells increased as stiffness increased, which were in agreement with some other recent studies [24, 55]. It has been reported that the adhesion ability of rMSSC-derived stem cells was increased on the higher stiffness of substrates, and cell–matrix adhesion was essential for cells metabolism, protein synthesis, and survival [56], and our results were also consistent with the findings of a previous study [49]. Furthermore, both osteogenic differentiation and vinculin expression were promoted on the stiffer substrates in our research, and an enhancement of vinculin has been reported to upregulate osteogenic differentiation of MSCs [57]. Therefore, our results underline the importance of further studying involvement of vinculin in stiffness-dependent cellular differentiation.

There are also several limitations associated with our study. First, given that other osteogenic-related cells also reside in maxillary sinus, such as osteoblasts and osteoclasts, the possible crosstalk of rMSSC-derived stem cells with these cells during the process of osteogenesis needs to be illustrated. Second, rMSSC-derived stem cells in this study were isolated only from rabbits, the antibody of rabbits is rare on the market, and so we only stained OPN for immunofluorescence images. Also, rabbit stem cells may have some differences with the human stem cells and hence further work in human cells is required. Third, Alizarin Red staining was used to detect the deposition of late osteogenic calcium salt. We did the alizarin red staining test, but there was no significant difference between the groups. Perhaps may be that the time of osteogenesis is short, only changes of early osteogenesis markers could be detected in our study. Fourth, although the stiffness could mimic extracellular physical signals, it might not represent a real extracellular microenvironment, which is complex due to existence of various factors like microtopography and chemical components. The fifth limitation is that structures of 2D substrates in our study were markedly distinct from the complicated structures in vivo. Better cell-culture platforms, including 3D systems with tunable stiffness, may overcome this disadvantage. Sixth, the signaling pathway associated with mechanotransduction in the regulation of stiffness-mediated differentiation of MSSC-derived stem cells should also be evaluated in future studies. In summary, substrate stiffness could significantly alter the behaviors of rMSSC-derived stem cells, and thus this factor should be carefully considered in the material design applied in maxillary sinus augmentation.

## Figures and Tables

**Table 1:** qRT-PCR primer sequences.

mRNA	Forward primer	Reverse primer
GAPDH	5′-TCACCATCTTCCAGGAGCGA	5′-CACAATGCCGAAGTGGTCGT
Runx2	5′-TCAGGCATGTCCCTCGGTAT	5′-TGGCAGGTAGGTATGGTAGTGG
OPN	5′-CACCATGAGAATCGCCGT	5′-CGTGACTTTGGGTTTCTACGC
COL-I	5′-CTTCTGGCCCTGCTGGAAAGGATG	5′-CCCGGATACAGGTTTCGCCAGTA
ALP	5′-CATCTCCCCTCTGGAACTCA	5′-CCAAACAGGAGAGTCGCT
BMP-2	5′-CGTGAGGATTAGCAGGTCTTTG	5′-CGCTTGACGCTTTTCTCTTCT

## Data Availability

The authors declare that all data of this study are available from the corresponding author upon request.
